# Cardiac Function in 7-8-Year-Old Offspring of Women with Type 1 Diabetes

**DOI:** 10.1155/2011/564316

**Published:** 2011-11-15

**Authors:** Maarten Rijpert, Johannes M. P. J. Breur, Inge M. Evers, Harold W. de Valk, Cobi J. Heijnen, Folkert J. Meijboom, Gerard H. A. Visser

**Affiliations:** ^1^Department of Pediatrics, Wilhelmina Children's Hospital, University Medical Center Utrecht, P.O. Box 85090, 3508 AB Utrecht, The Netherlands; ^2^Department of Pediatric Cardiology, Wilhelmina Children's Hospital, University Medical Center Utrecht, P.O. Box 85090, 3508 AB Utrecht, The Netherlands; ^3^Department of Obstetrics and Gynecology, Meander Medical Center, P.O. Box 1502, 3800 BM Amersfoort, The Netherlands; ^4^Department of Internal Medicine, University Medical Center Utrecht, P.O. Box 85500, 3508 GA Utrecht, The Netherlands

## Abstract

Offspring of type 1 diabetic mothers (ODMs) are at risk of short-term and long-term complications, such as neonatal macrosomia (birth weight >90th percentile), hypertrophic cardiomyopathy, and cardiovascular morbidity in later life. However, no studies have been performed regarding cardiac outcome. In this study, we investigated cardiac dimensions and function in 30 ODMs at 7-8 years of age in relation to neonatal macrosomia and maternal glycemic control during pregnancy and compared these with those in a control group of 30 children of nondiabetic women. We found that cardiac dimensions and systolic and diastolic function parameters in ODMs were comparable with those in controls. Neonatal macrosomia and poorer maternal glycemic control during pregnancy were not related to worse cardiac outcome in ODM. We conclude that cardiac function at 7-8 years of age in offspring of women with type 1 diabetes is reassuring and comparable with that in controls.

## 1. Introduction

Despite good prepregnancy care and adequate maternal glycemic control during type 1 diabetic pregnancies, the risk of perinatal complications in the offspring, such as preterm birth and neonatal macrosomia (birth weight >90th percentile), is still high compared with the general population [1]. Furthermore, there is increasing evidence that children born after a diabetic pregnancy are at increased risk of cardiovascular and metabolic morbidity at later age [2], especially when macrosomic at birth [3, 4]. 

Intrauterine hyperglycemia during type 1 diabetic pregnancy may lead to congenital heart defects, as has been shown in animal studies [5, 6]. In human studies, structural cardiac defects occur in 2–15% of newborn infants of type 1 diabetic women [1, 7–9]. Hypertrophic cardiomyopathy (HCM, mainly interventricular septal hypertrophy) can be demonstrated in 25–45% of offspring of type 1 diabetic women [9–13]. Interventricular septal hypertrophy may be associated with functional cardiac changes during pregnancy as well as in the neonatal period [13–18] and seems to normalize within the first six months after birth [12, 13]. Despite the fact that possible mechanisms regulating the development and resolution of neonatal HCM have been investigated in diabetic rats, they are still unknown [19, 20]. It is therefore not clear whether neonatal HCM may be important in terms of residual cardiac pathology at later age. However, to the best of our knowledge no follow-up studies regarding cardiac structure or function have been performed in offspring of women with type 1 diabetes at later age.

Because of the high prevalence of HCM in the neonatal period and the risk of later cardiovascular diseases in offspring of type 1 diabetic women, we hypothesized that cardiac dimensions and/or function may be altered at later age. The objectives of this study were to evaluate cardiac dimensions and function at school age in children who were born after a type 1 diabetic pregnancy in relation to neonatal macrosomia and maternal glycemic control during pregnancy and to compare these measurements to those in a control group of children of nondiabetic women.

## 2. Materials and Methods

### 2.1. Study Population

The study group consisted of offspring of type 1 diabetic mothers (ODMs) who participated in a previous nationwide study on type 1 diabetes and pregnancy outcome in The Netherlands [1]. We performed a follow-up study in 213 of these children at school age, which consisted of a home visit (for anthropometric measurements, blood pressure recordings, and neurocognitive tests) and a fasting blood sample on a separate occasion. More details of this cohort and results from anthropometric and cardiovascular measurements have been described elsewhere [21, 22]. ODM who participated in the follow-up study and lived within 50 kilometers of our hospital (*n* = 43) were invited for an additional echocardiogram, and 30 of them participated. Mean age of the ODM at time of the echocardiogram was 7.6 years (range 7.3–8.1). Information regarding maternal characteristics and pregnancy outcome was obtained from the previous study on pregnancy outcome [1], which had been provided by the attending gynecologist/internist. Information on neonatal outcome (including clinical diagnosis of HCM) had been provided by the attending pediatrician. Neonatal macrosomia was defined as birth weight >90th percentile for gestational age, sex, and parity according to the Netherlands Perinatal Registry data [23].

The control group of the original follow-up study consisted of randomly selected offspring of nondiabetic women without severe maternal disease during pregnancy, who were born in the same period as the ODM at the University Obstetric Center, Utrecht, The Netherlands (*N* = 79). In this center both low- and high-risk women from the province of Utrecht (from cities as well as from the countryside) deliver. From this control group we invited children based on order of inclusion to participate in an additional echocardiography study until we included 15 macrosomic and 15 nonmacrosomic controls.

Mean age of the controls at time of the echocardiogram was 7.4 years (range 6.9–8.1). Information regarding maternal characteristics and pregnancy outcome was obtained from hospital records and additional questionnaires. 

This study was approved by the Medical Ethics Committee of the University Medical Center Utrecht, The Netherlands. All parents gave written informed consent.

### 2.2. Measurements

During a home visit (previous to the echocardiography) blood pressure was recorded three times on the right arm in sitting position after five minutes of rest with a two-minute interval period, using an automated oscillometric device (DINAMAP, Critikon, Tampa, Fla). The average of the last two measurements of systolic (SBP) and diastolic (DBP) blood pressure was used for analysis. The children's height and weight were measured on the day of the echocardiography, and BMI was calculated. 

All participants underwent a full echocardiographic evaluation including a structural echo for cardiac defects and evaluation of systolic and diastolic left ventricular function. Diastolic dimensions of the left ventricle (LVEDd), interventricular septum (IVSd), and left ventricular posterior wall (LVPWd) were measured and Z-scores (corrected for height and weight) calculated [24]. Systolic left ventricular function was evaluated using shortening fraction (SF) and tissue Doppler imaging of the IVS and LVPW with measurement of IVS ′S and LVPW ′S (systolic peak wall motion velocity) [25–27]. Finally cardiac output (CO) was calculated per kilogram body weight as CO = (SV · HR) (with SV being stroke volume and HR being heart rate). SV was calculated as SV = LVOT_area_ · VTI(LVOT), with LVOT_area_ being left ventricular outflow tract area (*π* · (diameter/2)^2^) and VTI(LVOT) being velocity time integral of LVOT which was established through averaging three pulsed wave Doppler tracings in the LVOT. Systolic right ventricular function was evaluated based on measurement of the tricuspid annular plane systolic excursion (TAPSE). Maximal tricuspid regurgitation pressure gradient (TR max PG) was measured if present to estimate RV pressure. Diastolic left ventricular function was evaluated with pulsed wave Doppler signal of the mitral valve inflow pattern and pulmonary vein pattern [28]. E/A and S/D ratios were calculated (ratio of early/late left ventricular filling speed and ratio of systolic/diastolic pulmonary vein filling speed, resp.). Tissue Doppler imaging of the IVS and LVPW was performed with measurement of ′De (early diastolic peak wall motion velocity) and ′Da (late diastolic peak wall motion velocity) values. Finally, E/E′ ratios were calculated to estimate LV filling pressures [29]. 

All examinations were performed using a GE Vivid 7 Ultrasound Machine (GE Healthcare, UK). The echo technician was blinded for the origin of the participants (ODM or controls).

### 2.3. Statistical Methods

General characteristics and differences in measurements between ODM and controls and between subgroups of ODM were compared using independent *t*-test for normally distributed variables, Mann-Whitney *U* test for not normally distributed variables, and *χ* ^2^-test (or Fisher's exact test if appropriate) for categorical variables. The relation between maternal HbA1c level during pregnancy and measurements in the offspring was evaluated using Pearson's (or Spearman's, if appropriate) correlation coefficients. Data were analyzed using SPSS 15.0 for Windows (SPSS, Chicago, Ill). A *P* value <0.05 was considered to be statistically significant.

## 3. Results and Discussion

### 3.1. General Characteristics

Participating mothers in the ODM group (*n* = 30) only differed significantly from the nonparticipating mothers (i.e., women who had participated in the previous nationwide study on pregnancy outcome but had not participated in this study, *n* = 283) regarding parity (70.0% versus 49.8% nulliparous women, *P* = 0.04). All other maternal and neonatal characteristics did not significantly differ between the participating and nonparticipating ODM group.

In the participating ODM group, maternal mean age at delivery was significantly lower compared with that in the control group, and the percentage of nulliparous women was significantly higher (Table 1). Mean gestational age at delivery and mean birth weight were lower in ODM compared with controls. The macrosomic and appropriate-for-date ODM subgroups did not significantly differ regarding maternal or child characteristics, except for a higher mean birth weight in macrosomic ODM (3835 (3459–4210) grams versus 3132 (2748–3495) grams, *P* < 0.01). Neonatal HCM was diagnosed in three children from the ODM group (two boys and one girl) [1]. Maternal and child characteristics of these three children did not significantly differ from the rest of the ODM group. Mean systolic blood pressure in ODM was slightly higher compared with controls, but the difference did not reach significance (Table 1).

### 3.2. Echocardiography

**Figure 1:**
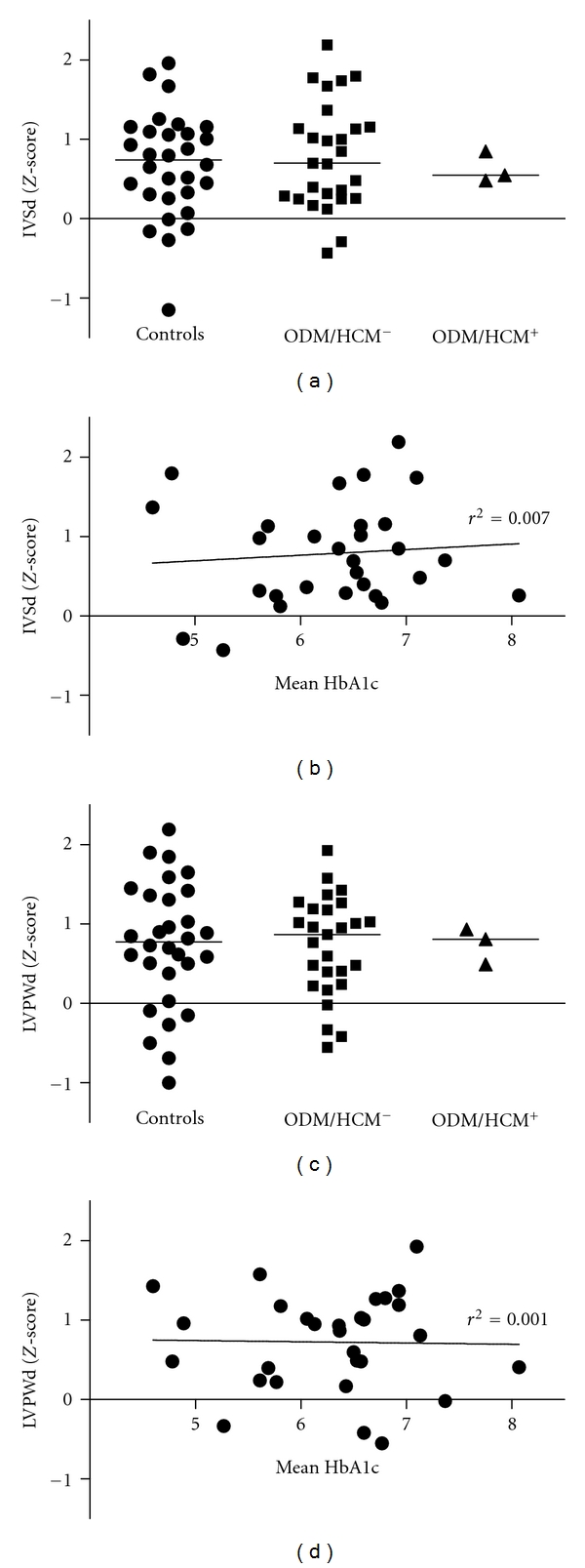
Cardiac dimensions in ODM regarding (a) neonatal hypertrophic cardiomyopathy and (b) maternal glycemic control during pregnancy (mean HbA1c level). ODM/HCM+ and ODM/HCM−: offspring of type 1 diabetic mothers with (+) or without (−) neonatal hypertrophic cardiomyopathy.

There were no significant differences in cardiac dimensions or systolic and diastolic cardiac function parameters between ODM and controls at 7 years of age (Table 2). All cardiac dimensions and function parameters in the three children with neonatal HCM were within the normal range (Figure 1(a)) and did not significantly differ from the other ODM or from controls. Subgroup analyses showed no significant differences in cardiac function or dimensions between macrosomic and appropriate-for-date ODM or between macrosomic ODM and macrosomic controls (Table 3). 

Echocardiographic measurements in ODM did not significantly correlate with maternal glycemic control during pregnancy (assessed by mean HbA1c level during first, second, and third trimester and mean HbA1c level during pregnancy; see also Figure 1(b)).

## 4. Discussion

Since (subclinical) HCM can be demonstrated in up to 45% of ODM and long-term cardiovascular sequelae in offspring born after a type 1 diabetic pregnancy may already present in childhood [2], we hypothesized that subtle changes in cardiac dimensions or function might also be present in ODM at school age. In this study, we are the first to show that systolic and diastolic function as well as cardiac dimensions in ODM at 7-8 years of age are completely normal and comparable with those in a control group of nondiabetic women. 

In our cohort of ODM only three children (out of 30) were diagnosed with HCM after birth. However, in newborn ODM from our cohort echocardiography was only performed when HCM was clinically suspected, in contrast to the studies reporting higher prevalences of neonatal HCM [9–13]. As fetal cardiac growth is promoted by binding of insulin to the cardiac insulin-like-growth-factor- (IGF-) 1 receptor, HCM is believed to resolve within weeks after birth due to normalization of fetal hyperinsulinaemia [30]. Indeed cardiac dimensions and function parameters in the three children with previous neonatal HCM were normal at 7 years of age, but a larger prospective follow-up study of ODM with HCM should substantiate these results. 

We previously showed that systolic blood pressure was significantly higher in ODM compared with controls [22]. In this subgroup of children who underwent additional echocardiography, the difference in systolic blood pressure did not reach statistical significance, most likely due to the fact that the groups were smaller. Despite a slightly higher mean SBP in ODM there were no differences in left ventricular function parameters between controls and ODM jet. Larger follow-up studies are necessary to investigate whether the difference in systolic blood pressure persists throughout childhood and may have consequences for cardiac function in later life. 

Previous studies have shown that offspring of diabetic women who were macrosomic at birth are at increased risk of developing overweight and other cardiovascular risk factors [3, 4]. Therefore, we investigated the possible influence of neonatal macrosomia on cardiac outcome in ODM. We found that neither cardiac dimensions nor cardiac function significantly differed between macrosomic ODM and those with an appropriate-for-date birth weight. As some studies have shown more cardiac alterations in macrosomic newborns of diabetic mothers when compared with macrosomic newborns of nondiabetic mothers [31, 32], we compared cardiac outcome of macrosomic ODM with that of macrosomic controls. No significant differences between those subgroups were found, indicating that neonatal macrosomia in ODM has no adverse effects on cardiac function at 7-8 years of age. 

Extrapolating Freinkel's theory on “fuel-mediated teratogenesis,” which states that high glucose concentrations during diabetic pregnancy make the developing tissues in the offspring vulnerable to alterations later in life [33], one might expect less favorable cardiovascular outcome in offspring of diabetic mothers with poorer glycemic control during pregnancy. However, maternal glycemic control during pregnancy did not significantly correlate with cardiac dimensions or function parameters in ODM at 7-8 years of age. It should be noted that maternal HbA1c may not be an accurate tool for the classification of level of glycemic control as it does not reflect the complexities of glycemic control in pregnant diabetic women [34]. 

As we are the first to describe cardiac dimensions and function in ODM at school age, this study should be a valuable addition to previous studies on long-term effects of a diabetic pregnancy on the development in the offspring. A limitation of this study is the relatively small sample size. Larger, ideally prospective follow-up studies should substantiate our results.

## 5. Conclusions

Cardiac function at 7-8 years of age in offspring of type 1 diabetic women is reassuring and comparable with that in children of nondiabetic women. Neonatal macrosomia and poorer maternal glycemic control during pregnancy were not related to adverse cardiac outcome in ODM.

## Figures and Tables

**Table 1:** Maternal and child characteristics in ODM and controls.

	Controls	ODM	*P*
*n*	30	30	
*Maternal characteristics*			
Age at delivery (years)	33.8 ± 3.0	31.4 ± 3.9	0.01
Parity (nulliparity)	10 (33.3)	21 (70.0)	<0.01
Race: Caucasian	30 (100)	29 (96.7)	1.0

*Pregnancy characteristics*			
Maternal smoking	1 (3.3)	2 (6.7)	1.0
Preeclampsia	2 (6.7)	4 (13.3)	0.7
Mean HbA1c 1st trim. (%)	—	6.51 ± 0.74	—
Mean HbA1c 2nd trim. (%)	—	6.04 ± 0.85	—
Mean HbA1c 3rd trim. (%)	—	6.32 ± 0.93	—

*Child characteristics*			
Gestational age (days)	277 [270–284]	260 [246–268]	<0.01
Birth weight (grams)	3793 ± 631	3400 ± 646	0.02
Birth weight percentile	87 [20–95]	84 [50–98]	0.2
Neonatal macrosomia	15 (50.0)	14 (46.7)	0.8
Sex (male)	16 (53.3)	14 (46.7)	0.6
Congenital malformation	0 (0)	1 (3.3)^a^	1.0
HCM at birth	0 (0)	3 (10.0)	0.2
Height at age 7 (cm)	131.0 ± 5.7	129.7 ± 5.9	0.4
BMI at age 7 (kg/m^2^)	15.3 [14.9–16.9]	16.3 [15.5–17.6]	0.1
SBP at age 7 (mmHg)	95.5 [91–100]	97 [94–105]	0.09
DBP at age 7 (mmHg)	58.0 ± 4.0	58.1 ± 5.0	0.9

Data represent mean ± standard deviation, median with interquartile range, or number with percentage. ^a^Single umbilical artery. Trim.: trimester; HCM: hypertrophic cardiomyopathy; SBP: systolic blood pressure; DBP: diastolic blood pressure.

**Table 2:** Echocardiographic measurements in ODM and controls at 7-8 years of age.

	Controls	ODM	*P*
*n*	30	30	
*Dimensions*			
IVSd (Z-score)	0.74 [0.30–1.12]	0.70 [0.28–1.15]	0.9
LVPWd (Z-score)	0.78 [0.29–1.38]	0.84 [0.36–1.18]	0.8
LVEDd (Z-score)	0.05 [−0.53–0.27]	−0.36 [−0.80–0.22]	0.2

*Systolic LV function*			
SF (%)	34.6 ± 4.9	35.3 ± 4.4	0.6
CO (mL/min/kg)	116 [102–134]	117 [99–131]	0.8
IVS ′S (cm/s)	7.4 [7.0–8.0]	7.4 [7.0–8.1]	0.5
LVPW ′S (cm/s)	10.6 [9.6–11.6]	10.0 [8.5–11.4]	0.3

*Systolic RV function*			
TAPSE (cm)	1.99 [1.85–2.21]	1.98 [1.86–2.17]	0.9
TR max PG (mmHg)	15.0 [14.1–17.0]	15.8 [13.1–17.3]	0.7

*Diastolic LV function*			
E/A ratio	2.2 [2.0–2.37]	2.14 [1.74–2.75]	0.9
S/D ratio	0.83 ± 0.27	0.83 ± 0.25	1.0
E/E′ ratio	6.1 [5.4–6.8]	6.3 [5.7–7.3]	0.4
MV DecT (ms)	175.5 ± 30.6	173.1 ± 33.7	0.8
IVS ′De (cm/s)	13.2 [12.7–14.4]	13.3 [12.0–14.9]	0.7
IVS ′Da (cm/s)	5.5 [5.0–6.5]	6.0 [5.4–6.1]	0.5
LVPW ′De (cm/s)	17.9 [15.6–19.7]	18.2 [16.5–19.0]	0.7
LVPW ′Da (cm/s)	6.2 [5.1–7.0]	6.7 [5.7–7.5]	0.2

Data represent means ± standard deviation or median with interquartile range. IVSd: interventricular septal end diastolic dimension; LVPWd: left ventricular posterior wall end diastolic dimension; LVEDd: left ventricular end diastolic dimension; SF: shortening fraction; CO: cardiac output; TAPSE: tricuspid annular plane systolic excursion; TR max PG: maximum tricuspid regurgitation pressure gradient; E/A: ratio of early and late left ventricular filling speed; S/D: ratio of systolic and diastolic pulmonary vein filling speed; E/E′ ratio: ratio of early diastolic peak E to E′ velocity; MV DecT: mitral valve deceleration time; ′S, ′De, ′Da: peak wall motion velocity during systole, early diastole or late diastole.

**Table 3:** Echocardiographic measurements in macrosomic ODM, appropriate-for-dates ODM, and macrosomic controls at 7-8 years of age.

	ODM	ODM		Controls	
	BW > p90	BW ≤ p90	*P* ^a^	BW > p90	*P* ^b^
*n*	14	16		15	
Age at echo (years)	7.6 [7.4–7.8]	7.6 [7.4–7.8]	0.7	7.6 [7.2–7.8]	0.7
BMI (kg/m^2^)	16.6 [16.0–17.7]	16.0 [15.2–17.0]	0.2	15.3 [15.1–16.9]	0.1

*Dimensions*					
IVSd (Z-score)	0.85 [0.31–1.29]	0.52 [0.25–1.11]	0.4	0.65 [0.07–1.10]	0.4
LVPWd (Z-score)	0.68 [0.21–1.21]	0.88 [0.48–1.14]	0.6	0.73 [0.03–1.03]	1.0
LVEDd (Z-score)	−0.37 [−0.60–0.25]	−0.35 [−1.22–0.25]	0.5	0.16 [−0.31–0.58]	0.2

*Systolic LV function*					
SF (%)	34.4 [31.4–37.7]	35.3 [33.5–38.7]	0.5	36.2 [33.9–41.8]	0.3
CO (mL/min/kg)	117 [113–126]	105 [102–136]	0.1	114 [100–133]	0.8
IVS ′S (cm/s)	7.2 [7.0–8.0]	8.0 [7.0–8.2]	0.6	8.0 [7.4–8.0]	0.4
LVPW ′S (cm/s)	9.8 [8.1–11.2]	10.0 [9.0–11.4]	0.6	10.7 [9.7–12.0]	0.4

*Systolic RV function*					
TAPSE (cm)	2.0 [1.8–2.3]	2.0 [1.9–2.1]	0.9	2.0 [1.9–2.1]	1.0
TR max PG (mmHg)	15.7 [13.1–17.3]	15.8 [13.3–17.4]	0.8	15.8 [14.5–18.2]	1.0

*Diastolic LV function*					
E/A ratio	2.1 [1.6–2.7]	2.1 [1.8–2.9]	0.4	2.1 [2.0–2.5]	1.0
S/D ratio	0.8 [0.7–1.0]	0.8 [0.6–0.9]	0.7	0.8 [0.7–1.0]	0.9
E/E′ ratio	6.1 [5.1–7.1]	6.5 [5.8–7.4]	0.7	5.9 [5.3–6.5]	0.6
MV DecT (ms)	175 [149–205]	176 [162–189]	0.8	176 [158–210]	0.6
IVS ′De (cm/s)	13.3 [12.0–15.0]	13.3 [11.8–14.5]	0.7	14.0 [13.0–15.0]	0.4
IVS ′Da (cm/s)	6.0 [5.5–6.0]	6.0 [5.4–6.3]	0.9	6.0 [5.0–7.0]	1.0
LVPW ′De (cm/s)	19.0 [18.2–20.7]	17.8 [16.3–18.8]	0.1	18.4 [17.0–20.0]	0.6
LVPW ′Da (cm/s)	6.2 [5.7–7.1]	7.0 [5.8–7.5]	0.6	6.3 [5.5–7.9]	1.0

Data represent median with interquartile range. ^a^Macrosomic ODM versus appropriate-for-date ODM. ^b^Macrosomic ODM versus macrosomic controls. BW: birth weight.

## Abbreviations

BMI: Body mass index
CO: Cardiac output
′Da: Peak wall motion velocity during late diastole (atrial contraction)
DBP: Diastolic blood pressure
′De: Peak wall motion velocity during early diastole
E/A: Ratio of early and late (atrial contraction) left ventricular filling speed
E/E′ ratio: Ratio of early diastolic peak E to E′ velocity
IVS: Interventricular septum
IVSd: Interventricular septal end diastolic dimension
LVEDd: Left ventricular end diastolic dimension
LVPW: Left ventricular posterior wall
LVPWd: Left ventricular posterior wall end diastolic dimension
MV DecT: Mitral valve deceleration time
ODM: Offspring of type 1 diabetic mothers
′S: Peak wall motion velocity during systole
SBP: Systolic blood pressure
S/D: Ratio of systolic and diastolic pulmonary vein filling speed
SF: Shortening fraction
TAPSE: Tricuspid annular plane systolic excursion
TR max PG: Maximum tricuspid regurgitation pressure gradient.
